# Delay and inequalities in the treatment of idiopathic pulmonary fibrosis: the case of two Nordic countries

**DOI:** 10.1186/s40248-018-0126-7

**Published:** 2018-05-14

**Authors:** Ida Pesonen, Lisa Carlson, Nicola Murgia, Riitta Kaarteenaho, Carl Magnus Sköld, Marjukka Myllärniemi, Giovanni Ferrara

**Affiliations:** 1 Department of Respiratory Medicine and Allergy, Karolinska University Hospital, SE-17176 Stockholm, Sweden; 2 Respiratory Medicine Unit, Department of Medicine Solna, Karolinska Institutet, Stockholm, Sweden; 3 Section of Occupational Medicine, Respiratory Diseases and Toxicology, University of Perugia, Perugia, Italy; 4 Department of Occupational and Environmental Medicine, University of Gothenburg, Gothenburg, Sweden; 5 Research Unit of Internal Medicine, University of Oulu and Medical Research Center Oulu, Oulu University Hospital, Oulu, Finland; 6 University of Helsinki and Helsinki University Hospital, Heart and Lung Center, Helsinki, Finland

**Keywords:** Idiopathic pulmonary fibrosis, Interstitial lung disease, Nintedanib, Pirfenidone, Access to healthcare

## Abstract

**Background:**

Idiopathic pulmonary fibrosis (IPF) is characterized by progressive loss of lung function with high mortality within the first 5 years from diagnosis. In 2011–2014, two drugs, pirfenidone and nintedanib, have been approved worldwide for prevention of IPF progression. National IPF-registries have been established in both Finland and Sweden. Our study explored potential differences in the care of IPF in these two countries.

**Methods:**

Patients included consecutively in the Finnish and Swedish IPF-registries from January 1, 2014 through December 31, 2016 were included in the study. Data on demographics and lung function at the time of inclusion were collected. Access to antifibrotic drugs and data on disease outcomes, mortality and the proportion of patients who underwent lung transplantation, was collected during a 3-year follow up.

**Results:**

One-hundred and fifty-two patients from the Finnish and 160 patients from the Swedish IPF-cohorts were included in the study. At inclusion, Finnish patients were significantly older than the Swedish patients (74.6 years vs 72.5 years, *p* = 0.017). The proportion of non-smokers was significantly higher in the Finnish cohort (41.7% vs 26.9%, *p* = 0.007). Forced vital capacity (FVC), % of predicted (78.2 vs 71.7 for Finnish and Swedish patients, respectively, *p* = 0.01) and diffusion capacity for carbon monoxide (DL_CO_), % of predicted (53.3 vs 48.2 for Finnish and Swedish patients, respectively, *p* = 0.002) were significantly higher in the Finnish cohort compared to the Swedish cohort at the time of inclusion. During the 3-year follow up period, 45 (29.6%) Finnish and 111 (69.4%) Swedish patients, respectively, were initiated on treatment with an antifibrotic drug (pirfenidone or nintedanib) (*p* <  0.001). When comparing possible determinants of treatment, patients with higher FVC % were less likely to start antifibrotic drugs (OR 0.96, 95%CI 0.93–1.00, *p* <  0.024). To be resident in Sweden was the main determinant for receiving antifibrotic drugs (OR 5.48, 95%CI 2.65–11.33, *p* < 0.0001). No significant difference in number of deaths and lung transplantation during the follow up period was found.

**Conclusions:**

This study highlights differences concerning how IPF patients are treated in Finland and Sweden. How these differences will influence the long-term outcome of these patients is unknown.

## Background

Idiopathic pulmonary fibrosis (IPF) is the most common type of the idiopathic interstitial pneumonias (IIP) characterized by a progressive fibrosis and loss of lung function [1]. Idiopathic pulmonary fibrosis is diagnosed by a typical radiological finding of usual interstitial pneumonia (UIP) on high resolution computed tomography (HRCT) or, in some cases, by a histological investigation of lung biopsy [1]. Two drugs (pirfenidone and nintedanib) have shown to slow down the decline of lung function in IPF patients, and combined treatments are currently under clinical investigation [2, 3]. In addition to drug treatment, early integration of supportive care and possible lung transplant evaluation are recommended [4, 5].

During the last few years, national registries collecting clinical data from IPF patients have been established [6–9]. These registries enable prospective follow up of real-life IPF patients’ disease course. The IPF registry (FinnishIPF) in Finland was started in 2011 [10] and currently includes nearly 700 patients. Previous studies on the Finnish IPF registry revealed that Finnish IPF patients are diagnosed at an early stage of the disease with mild or moderate loss of lung function [11]. The Swedish IPF registry was started in 2014 and data on diagnostic evaluations, demographics, lung function, laboratory tests and quality of life have been successfully collected since then [7].

There are no previous comparative studies on the real-life clinical presentation and management of IPF in the Nordic countries. Finland and Sweden are neighboring countries with many similarities. Furthermore, there is a close collaboration between the countries and specialists from the main university hospitals, and a shared position paper on the diagnosis and treatment of IPF was recently published as a first attempt to optimize and uniform health care for IPF patients in the Nordic countries [12].

This cohort study was performed to assess whether IPF shares common presentation in two neighboring European countries, and whether patients have the same opportunities in terms of access to health care and treatment. We compared the main clinical presentation, demographics, lung function and access to specific treatments in Finland and Sweden using the respective national registries as source of data.

## Methods

### The National IPF registries

The Finnish IPF registry was created in 2011 on a web-based platform (Granitics Unify Med, Granitics Ltd., Espoo, Finland) and collects today patients from 27 different hospitals across the country. Inclusion criteria to the registry are patients diagnosed with IPF [10]. In 2014, the same platform was chosen and adapted for the Swedish data collection to become the Swedish IPF registry [7]. Patients are currently enrolled from 22 different hospitals across Sweden. The inclusion of IPF patients in both registries is based on the fulfillment of the main international diagnostic criteria [1], and informed consent is signed in both countries upon inclusion.

Demographics, clinical data such as lung function, comorbidities, radiology, histopathology, prescribed treatments and outcomes are reported in both registries [7, 13]. All these variables are duly updated in both registries by the investigators at site and by two research nurses in the respective headquarters (Helsinki in Finland and Stockholm in Sweden) at the time of inclusion and after every clinical visit usually between every 3–6 months.

For the aims of this study, only patients included in the two registries with a diagnosis of IPF from January 1, 2014 to December 31, 2016 were considered. Patients who received another diagnosis than IPF during the course of their disease were excluded from this study. Furthermore, patients treated with antifibrotic drugs before January 1, 2014 were excluded from the study.

### Data collection

Data on age, sex, body mass index (BMI), smoking habits (smoking status, pack/years), forced vital capacity (FVC, liters and % of predicted), forced expiratory volume (FEV_1_, liters and % of predicted) and diffusing capacity for carbon monoxide (DL_CO_, % of predicted) at the time of inclusion were extracted from the registries. Percent of predicted values for lung function were reported with the Finnish reference values from 1982 [14] in the Finnish registry and with the Swedish reference values in the Swedish registry [15, 16].

Follow up-data were collected during the study period (January 1, 2014 - December 31, 2016). Information regarding the prescription of antifibrotic treatments and the time between inclusion and start of treatment (weeks) were collected during this period, as well as data on outcomes (death and lung transplantation).

### Statistical analysis

Gender distribution and smoking habits were presented as proportions and compared with Chi-squared test between the Finnish and Swedish cohorts. Data on age, sex, BMI, smoking habit, FVC, FVC %, FEV_1_, FEV_1_%, DL_CO_ % and time from inclusion to treatment with antifibrotic drugs were presented as mean and standard deviation (SD). Parametric and non-parametric statistical tests (Mann-Whitney test) were used when appropriate to compare means between the two cohorts.

The nominal data on prescribing a treatment with antifibrotic drugs during the study period (yes or no) was assessed as proportions for the two groups and compared with Chi-squared test. An univariate analysis was performed to assess differences between patients receiving and not receiving a treatment with antifibrotic drugs. To assess the influence of potential independent factors (gender, age, lung function, country, type of hospital) on the starting of treatment with antifibrotic drugs a logistic regression analysis was performed.

All the analyses were performed using the statistical software SPSS 20.0 (IBM Corp, Armonk, NY, USA).

## Results

### Registry population

One hundred fifty-eight eligible patients in the Finnish IPF registry and 174 patients in the Swedish IPF registry fulfilling the inclusion criteria were included in the study. Six patients were excluded from the Finnish cohort due to another ILD diagnosis during the follow up (two patients with rheumatoid arthritis, two patients with hypersensitivity pneumonitis, one with microscopic polyangitis and one with NSIP). Fourteen IPF patients from the Swedish cohort were excluded from the analysis because they were recently transferred to the registering center from other centers/specialists and were already on antifibrotic treatment before the inclusion. One hundred fifty-two and 160 patients from the Finnish and Swedish cohorts, respectively, were analyzed.

### Demographics and lung function

There were no differences in gender distribution and BMI between the cohorts (Table 1). However, Finnish patients were significantly older compared to the Swedish patients at inclusion, and the proportion of never smokers was significantly higher in the Finnish cohort (Table 1). Nine Finnish patients (5.9%) and 7 Swedish patients (4.4%) were current smokers (*p* = 0.54). Forced vital capacity, % of predicted and DL_CO_ % were significantly higher in the Finnish cohort compared to the Swedish cohort (Table 2).

**Table 1:** Demographics of the study population extracted from the Finnish and Swedish registries

Variable	N	FINLAND *N* = 152	SWEDEN *N* = 160	*p*
Gender				
Female *n* (%)	95	48 (31.6)	47 (29.4)	0.672
Male *n* (%)	217	104 (68.4)	113 (70.6)	
Age at inclusion mean (SD)	312	74.6 (8.3)	72.5 (8.0)	0.017
BMI^a^, kg/m^2^, mean (SD)	268	28.5 (5.5)	27.1 (4)	0.083
Smoking habits				
Never smoker *n* (%)	102	63 (41.7)	39 (26.9)	0.007
Ex or current smoker *n* (%)	195	88 (58.3)	106 (73.1)	
Pack/years mean (SD)	144	24.4 (13.7)	24 (14.8)	0.716

Definitions on abbreviations: ^a^*BMI* Body Mass Index; *SD* standard deviation

**Table 2:** Lung function at inclusion in the IPF patients extracted from the Finnish and Swedish registries

Variable	*N* 312	FINLAND *N* = 152	SWEDEN *N* = 160	*p*
FEV_1_^a^, % predicted, mean (SD)	288	77.7 (19.4)	77.2 (17.1)	0.817
FVC^b^, L, mean (SD)	278	2.71 (0.84)	2.76 (0.83)	0.614
FVC, % predicted, mean (SD)	283	78.2 (17.7)	71.7 (16)	0.010
DL_CO_^c^, % predicted, mean (SD)	238	53.3 (14.4)	48.2 (14.7)	0.002

Definitions on abbreviations: ^a^*FEV*_*1*_ Forced expiratory volume; ^b^*FVC* Forced vital capacity; ^c^*DL*_*CO*_ Diffusing capacity of carbon monoxide; *SD* standard deviation

### Treatment with antifibrotic drugs

During the follow up period, 45 (29.6%) Finnish and 111 (69.4%) Swedish patients, respectively, were prescribed an antifibrotic drug (pirfenidone or nintedanib, *p* < 0.001). The mean time from inclusion to drug treatment was significantly longer in Finland than in Sweden (26±31.3 vs 5.3±14.4 weeks, *p* < 0.001). Younger patients were more likely to receive antifibrotic drug treatment compared to older patients (Table 3). Patients in the drug treatment group had also significantly lower FVC % and DL_CO_ % compared to the non-treatment group (Table 3).

**Table 3:** Demographics and clinical features of the patients divided for treatment with antifibrotic drugs

Variable	*n*	TREATMENT		*p*
		NO *N* = 156	YES N = 156	
Gender				
Female *n* (%)	95	56 (35.9)	39 (25)	0.036
Male *n* (%)	217	100 (64.1)	117 (75)	
Age at inclusion mean (SD)	312	74.9 (8.7)	72.1 (7.4)	0.001
BMI^a^, kg/m^2^, mean (SD)	268	27.3 (4.5)	28.1 (5.0)	0.393
Smoking habits				
Never smoker n (%)	102	58 (38.4)	54 (30.3)	0.144
Ex or current smoker n (%)	195	93 (61.6)	101 (69.7)	
Pack/years mean (SD)	144	24 (14.3)	24.2 (14)	0.851
Lung function tests				
FEV_1_^b^, L, mean (SD)	286	2.18 (0.71)	2.20 (0.60)	0.833
FEV_1_, % predicted, mean (SD)	288	80.1 (20.6)	75 (15.3)	0.040
FVC^c^, L, mean (SD)	278	2.75 (0.89)	2.73 (0.77)	0.844
FVC, % predicted, mean (SD)	283	79.3 (19.2)	70.5 (13.5)	< 0.001
DL_CO_^d^, % predicted, mean (SD)	238	54.7 (14.2)	47.3 (14.5)	< 0.001
Country				
Finland	152	107 (68.6)	45 (28.8)	< 0.001
Sweden	160	49 (31.4)	111 (71.2)	

Definitions on abbreviations: ^a^*BMI* Body Mass Index; ^b^*FEV*_*1*_ Forced expiratory volume; ^c^*FVC* Forced vital capacity; ^d^*DL*_*CO*_ Diffusing capacity of carbon monoxide; *SD* standard deviation

A multivariate analysis was performed to explore which factors were determinants of treatment with antifibrotic drugs. In particular, FVC % was inversely correlated with drug treatment (Table 4). The strongest determinant for starting a treatment was to be resident in Sweden, with an odds ratio of 5 times for Swedish patients compared to Finnish patients (Table 4). These results did not change when a sensitivity analysis was performed taking into account if the patients were treated at university or peripheral hospital (Table 4).

**Table 4:** Determinants of treatment with antifibrotic drugs, logistic regression analysis during the study period (January 2014 – December 2016)

Variable	ODDS RATIO	95%CI	*p*
Female gender	0.76	0.39–1.48	0.419
Age at inclusion	0.98	0.95–1.02	0.413
FVC^a^, % predicted	0.96	0.93–1.00	0.024
FEV_1_^b^, % predicted	1	0.97–1.04	0.744
DL_CO_^c^, % predicted	0.98	0.96–1.01	0.171
Country			
Finland	1		< 0.0001
Sweden	5.48	2.65–11.33	
Type of Hospital			
Peripheral	1		0.503
University	1.27	0.63–2.57	

Definitions on abbreviations: ^a^*FEV*_*1*_ Forced expiratory volume; ^b^*FVC* Forced vital capacity; ^c^*DL*_*CO*_ Diffusing capacity of carbon monoxide

No difference was found in the number of deaths, 34 (22.4%) and 26 (16.3%) among the Finnish and Swedish patients, respectively (*p* = 0.17). Neither was there any differences in the number of patients undergoing lung transplantation (1 each, 0.01% in both the Finnish and Swedish cohorts, *p* = 0.97) during the study period.

## Discussion

Our study demonstrates significant differences in the clinical characteristics and initiation of treatment with antifibrotic drugs in IPF in two neighboring Nordic countries based on those subjects included into the IPF-registries. Finnish IPF patients were older, had better lung function, were less likely to have smoked and were prescribed less antifibrotic drugs compared to Sweden.

The better lung function in Finnish patients at the point of inclusion may be a result of diagnosis at an earlier stage of the disease compared to Swedish patients. The proportion of ex- or current- smokers in both countries was lower than what was previously shown with e.g. Danish patients, where a cohort study showed that 81% of the patients were ex- or current- smokers [17].

A previous study showed that organization of specific services can vary among different Nordic countries and within countries, depending on resources and local guidelines [18]. Finland and Sweden are neighboring countries with many similarities but also marked differences in health care systems. It is shown that there are significant genetic differences between these two populations but also within Finland between subpopulations in Eastern and Western Finland [19, 20].

Our study shows that there are differences in how patients are treated with antifibrotic drugs in Finland and Sweden. Close to 30% of Finnish patients started treatment compared to nearly 70% of the patients in Sweden. Although nintedanib reached the markets in 2015, there was no difference in the availability of drugs in the two countries during the study period; pirfenidone was introduced in Sweden in 2011 and in Finland in 2013, way before our study started. However, nor of the registries have a full coverage of the IPF population in each country, and therefore the bias of selection of the patients may have some effect to the results. It is to note, anyway, that all the university hospitals and all the main peripheral hospitals are actively reporting patients in Finland and Sweden.

Therefore, we report a consistent and significant difference in the use of antifibrotic drugs in Finland and Sweden, not explained by the availability of the drugs on the market. Differences in reimbursement systems between these two countries could account for this difference. The prescription of drugs in Finland is regulated by the social insurance institution of Finland. Patients with IPF are required to have a FVC % value between 50 and 90% to receive the drugs at lower costs, but still need to pay an annual cost of over six hundred Euros. Furthermore, until 2015, the upper limit for FVC % was 80% which means that once reached the markets, antifibrotic drugs was not an option for patients with FVC % over 80% for at least two years. The reimbursement is applied for every patient and the processing time for applications can vary between 2 and 6 weeks. On the other hand, the antifibrotic drugs are included in the general high-cost protection in Sweden and, therefore, there is no need to apply for reimbursement, patients can purchase drugs as soon as a physician has made a decision on drug initiation. A Swedish patient pays no more than around 230 Euros for drugs entitled to high-cost protection within a year. Furthermore, since 2012, antifibrotic drugs can be prescribed in Sweden regardless of the lung function values.

Previous studies show benefits of treating patients with mild to moderate loss of lung function [5, 21, 22]. This study revealed common tendencies of drug prescription in both countries; Firstly, a lower FVC % seems to favor treatment, as it does a younger age. Limited studies on the treatment of patients at the extremes of lung function (well preserved or severely impaired) and regional guidelines (allocating resources and priorities at a local level) are probably two of many reasons affecting the decision to treat with antifibrotic drugs. However, recent studies show that both patients with preserved lung function and with a severe loss of lung function may benefit from treatment [23–25].

During the study period, no significant difference in disease outcomes was shown. However, we consider the period as too short and the cohort size as too small to make any conclusions; a longer follow up period and larger cohort sizes are needed to show some potential differences in either lung function decline or mortality, even in consideration of the differences in the prescription of the treatment.

There are some possible limitations when working with two independent registries which could contribute to the differences. We cannot exclude a bias in the recruitment of patients to the registries in the two countries, i.e. patients could be recruited in Sweden mostly in university- or reference centers and they could be more prone to be treated than in peripheral hospitals in Finland. On the other hand, the comparison between the university and peripheral hospitals revealed no difference for what concerns treatment with antifibrotic drugs between these two cohorts.

Finland and Sweden are using different, local reference values for the lung function tests which could contribute to the differences in the observed lung function in per cent of predicted. However, we investigated in this study which factors did support the decision to treat with antifibrotic drugs in real-life practice in the two countries; the real-life reported value of the FVC % of the predicted value was the main index used by physicians in both countries, and our study clearly shows that patients in Finland and Sweden are treated when a big proportion of lung function is already lost. These findings should stimulate the discussion about the need of an early diagnosis and treatment of IPF.

None of the patients included in the Finnish and Swedish registries were enrolled in ongoing clinical trials during the study period. This is an important information, as the participation of clinical centers in ongoing trials could have potentially increased the number of patients receiving drugs, regardless of local regulations.

## Conclusions

This study shows a unique comparative cohort of modern, high-quality registry data from Finland and Sweden. Even though these health care systems can be evaluated as being very similar with predominating public health care systems, especially for rare diseases, we were able to pinpoint marked differences in the IPF patients’ access to antifibrotic therapy. In addition, we were able to identify differences in population characteristics, namely patient's smoking habits and age. Data from both countries suggests that physicians tend to prescribe antifibrotic drugs to patients that are younger and have a more advanced disease. Further studies are needed to assess how the inequality in treatment will affect long-term outcomes such as lung function, mortality and transplantation rates.

## Abbreviations

BMI: Body Mass Index
Dl_CO_%: Diffusing Capacity of Carbon Monoxide, % of predicted
FEV_1_: Forced expiratory volume
FEV_1_%: Forced expiratory volume, % of predicted
FVC %: Forced vital capacity, % of predicted
FVC: Forced vital capacity
HRCT: High resolution computed tomography
IIP: Idiopathic interstitial pneumonia
ILD: Interstitial lung disease
IPF: Idiopathic pulmonary fibrosis
NSIP: Non-specific interstitial pneumonia
UIP: Usual interstitial pneumonia
